# The SWI/SNF protein ATRX co-regulates pseudoautosomal genes that have translocated to autosomes in the mouse genome

**DOI:** 10.1186/1471-2164-9-468

**Published:** 2008-10-08

**Authors:** Michael A Levy, Andrew D Fernandes, Deanna C Tremblay, Claudia Seah, Nathalie G Bérubé

**Affiliations:** 1 Department of Biochemistry, University of Western Ontario, London, N6A 4L6, Canada; 2 Applied Mathematics, University of Western Ontario, London, N6A 4L6, Canada; 3 Paediatrics, University of Western Ontario, London, N6A 4L6, Canada; 4 Children's Health Research Institute, Lawson Health Research Institute, 800 Commissioners Road East, London, N6C 2V5, Canada

## Abstract

**Background:**

Pseudoautosomal regions (PAR1 and PAR2) in eutherians retain homologous regions between the X and Y chromosomes that play a critical role in the obligatory X-Y crossover during male meiosis. Genes that reside in the PAR1 are exceptional in that they are rich in repetitive sequences and undergo a very high rate of recombination. Remarkably, murine PAR1 homologs have translocated to various autosomes, reflecting the complex recombination history during the evolution of the mammalian X chromosome.

**Results:**

We now report that the SNF2-type chromatin remodeling protein ATRX controls the expression of eutherian ancestral PAR1 genes that have translocated to autosomes in the mouse. In addition, we have identified two potentially novel mouse PAR1 orthologs.

**Conclusion:**

We propose that the ancestral PAR1 genes share a common epigenetic environment that allows ATRX to control their expression.

## Background

The sex chromosomes in modern placental mammals (eutherians) are highly dimorphic but initially evolved from a homologous pair of autosomes [1]. Over millions of years of mammalian evolution, the sex chromosomes have lost most of their homology due to chromosome Y attrition [2]. The remaining homology between the sex chromosomes exists in the pseudoautosomal regions (PARs), located at the ends of the X and Y chromosomes [3] and was generated when genetic material from the tips of autosomes translocated to the ancient sex chromosomes [4]. Gene dosage between XX females and XY males is usually achieved by the silencing of one X chromosome in every female cell, a process known as X chromosome inactivation (XCI) [5]. Because both males and females have two copies of all PAR genes there is no requirement for dosage compensation and these genes therefore escape this inactivation process [6].

**Figure 1:**
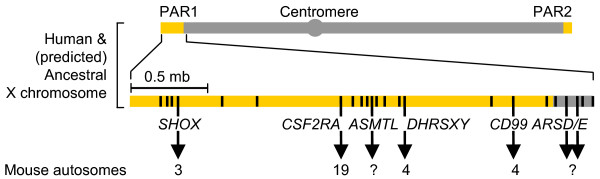
**Evolution of PAR genes in humans and mice**. PAR genes that are downregulated in the ATRX-null mouse forebrain are clustered together within the PAR1 region of common evolutionary ancestors of humans and mice but have translocated to autosomes in the mouse. Vertical lines and arrows represent individual genes. The position of the first nucleotide of each gene is as follows: *SHOX *(505,079), *CSF2RA *(1,347,701), *ASMTL *(1,482,032), *DHRSXY *(2,147,553), *CD99 *(2,619,553), *ARSD *(2,848,421), *ARSE *(2,832,011) (Human reference sequence NCBI Build 36.1). PAR1, pseudoautosomal region 1; PAR2, pseudoautosomal region 2. PAR regions are highlighted in orange.

Comparison of human PARs with those of other primates, carnivores (dogs and cats) and artiodactyls (cattle, sheep, pigs; representing the common evolutionary ancestor between humans and mice) has revealed that gene content is mostly conserved in eutherians, including the existence of PARs at both ends of the X and Y chromosomes [4]. However, rodents are strikingly different in that they have a single, dissimilar and considerably shorter PAR region [7]. Fewer than half of the 24 PAR1 genes identified so far in humans have also been found in the mouse genome, and all have diverged considerably [7]. This divergence is largely due to the increased recombination rates in the PARs during male meiosis [8]. In addition, the PARs comprise a unique chromosomal environment that is rich in repetitive sequences [9,10]. For these reasons, the identification of human PAR genes and orthologs has been difficult. Interestingly, in the mouse, all human PAR1 orthologs identified to date are located on autosomes. For example, colony stimulating factor 2 receptor, alpha (*Csf2ra*) is located on mouse chromosome 19 [11] and CD99 antigen (*Cd99*) and Dehydrogenase/reductase short-chain dehydrogenase/reductase family, X chromosome (*Dhrsxy*) are located on chromosome 4 [9,12]. Human orthologs of acetylserotonin O-methyltransferase-like (*ASMTL*) and several members of the arylsulfatase (ARS) family of genes (*ARSE*, *ARSD*, *ARSF*, and *ARSH*) located just outside the PAR1, have not yet been reported in the mouse (Figure 1). Due to their location in the PAR region of evolutionary ancestors, and their current autosomal location, we will refer to these genes in the mouse as "ancestral PAR genes".

The α thalassemia mental retardation, X linked (ATRX) protein, transcribed from Xq13.3 belongs to the Sucrose non-fermenting 2 (Snf2) family of enzymes that use the energy of adenosine tri-phosphate (ATP) hydrolysis to disrupt nucleosome stability [13,14]. Mutations in *ATRX *result in moderate to profound cognitive deficits, facial dysmorphisms, as well as skeletal and urogenital abnormalities, among other symptoms [15]. The chromatin remodeling properties of ATRX have been demonstrated *in vitro *[16]. In addition to a conserved ATPase/helicase domain, ATRX has an N-terminal zinc finger ATRX-DNMT3A/B-DNMT3L (ADD) domain that is shared with *de novo *methyltransferases. Several lines of evidence have also linked ATRX to highly repetitive genomic regions including pericentromeric heterochromatin in mouse and human cells [17]. Moreover, *ATRX *mutations in humans result in aberrant DNA methylation patterns at several repetitive elements, including ribosomal DNA (rDNA) repeats, subtelomeric repeats and Y-specific satellite repeats [18]. These repetitive sequences usually form heterochromatic structures and seem to be specifically targeted by the ATRX protein.

To assess the role of ATRX in brain development, we previously used Cre-*lox*P recombination to remove *Atrx *specifically in the forebrain beginning at E8.5. Loss of ATRX in the embryonic forebrain caused hypocellularity and a reduction in forebrain size and loss of the dentate gyrus [19].

Genes that are directly regulated by the ATRX protein have not yet been identified in either humans or mice. To identify potential genes that are controlled by ATRX, we performed a screen of gene expression and found that a subset of ancestral PAR1 genes is consistently downregulated in the absence of ATRX in the developing mouse brain. Among them are two potentially novel mouse orthologs of *Arse *and *Asmtl*. The only common link between ancestral PAR genes is their adjacent location and shared chromatin environment in the ancestral PAR region. We propose that conserved sequences and/or chromatin features targeted by ATRX were maintained upon translocation of these genes from the PAR1 on the ancestral X chromosome to their current location on mouse autosomes, and allow ATRX to modulate their expression.

## Results

### Effects of ATRX deletion on forebrain gene expression

The ability of ATRX to remodel chromatin [16] suggests that ATRX can regulate gene expression. To identify possible gene targets of the ATRX protein in the developing mouse brain, we used the previously described *Atrx*^*Foxg1Cre *^mice that lack ATRX in the forebrain [19]. In this model system, *Atrx *deletion is achieved by crossing *Atrx*^*loxP *^"floxed" mice to mice that express cyclization recombinase (Cre) under the control of the forebrain-specific forkhead box G1 (*Foxg1*) promoter [20]. We performed microarray analysis to compare the expression profiles of the *Atrx*^*Foxg1Cre *^and control telencephalon at embryonic day 13.5 (E13.5) (n = 3 pairs) using an Affymetrix mouse genome expression array representing approximately 39,000 transcripts [21]. Only probe sets showing a significant difference (p < 0.05) were included in all subsequent studies. By setting a threshold of 1.5 fold change we identified 202 disregulated probesets, and at a threshold of 2 fold change we identified only 22 altered probe sets. Approximately two-thirds of the probe sets demonstrating altered expression were upregulated (Additional file 1A, B).

We next compared gene expression patterns in control and *Atrx*-null forebrain tissue at postnatal day 0.5 (P0.5) (n = 4 pairs). At a threshold of 1.5 fold change, we identified 304 probe sets and at a threshold of 2 fold change, we identified 57 probe sets showing altered transcript levels. When we compared the microarray results at E13.5 and P0.5 we identified 14 common probe sets that were upregulated and 13 that were downregulated more than 1.5 fold, and one increased and three decreased more than 2 fold (Additional file 1A).

We used GeneSpring to identify significantly overrepresented Gene Ontology (GO) categories in the *Atrx*-null mouse forebrain. Several statistically and biologically significant categories of upregulated genes were related to the immune response. This could be an indirect response to the increased apoptosis that characterizes the *Atrx*-null forebrain at E13.5 in the developing cortex and to a lesser extent at P0.5 in the hippocampus [19]. In particular, categories and genes involved in phagocytotic clearing of apoptotic cells, such as complement activation [22], were enriched at both E13.5 and P0.5. Several genes involved in cell adhesion processes were upregulated at P0.5 and, consistent with the abnormal forebrain development described in the Atrx-null forebrain[19], genes involved in neurogenesis and nervous system development were downregulated at both timepoints (Additional file 2).

### Ancestral pseudoautosomal genes are downregulated in the Atrx-null mouse forebrain

Five of the most downregulated transcripts identified in the microarray analysis were unidentified cDNA clones (Affymetrix IDs 1436320_at, 1448057_at, 1443755_at, 1429730_at and 1453066_at; Accessions [GenBank:W45978], [GenBank:BI202412], [GenBank:BE457721], [GenBank:AK007409] and [GenBank:BI320076] respectively). To further investigate these probe sets, their NCBI nucleotide sequences were used for a Basic Local Alignment Search Tool nucleotide (BLASTn) search of the nr database. The expressed sequence tag (EST) [GenBank:W45978] has similarity to *Mus musculus Dhrsxy *([GenBank:NM_001033326], score = 120, E value 5e-24). The EST [GenBank:BI202412] displayed similarity to several unidentified mouse cDNA clones. Interestingly, a BLAST-like Alignment Tool (BLAT) search of this clone showed similarity to intron 1 of mouse *Dhrsxy *and it could represent an unknown splice variant of *Dhrsxy*. The EST [GenBank:BE457721] is annotated as similar to human *Arse *and a BLASTn search revealed high similarity to *Rattus norvegicus Arse *([GenBank:NM_001047885], score 197, E value 6e-28). BLASTn of [GenBank:AK007409] showed high similarity to *Asmtl *in cow ([GenBank:BT02626], score = 248, E value = 6e-62) as well as dog, human, the putative rat *Asmtl*, and numerous other species. The EST [GenBank:BI320076] displayed no significant hits to any sequences by either BLASTn or BLAT. Interestingly, while *Dhrsxy*, *Arse *and *Asmtl *do not display an obvious connection, they do share a common link in that they are all pseudoautosomal genes in eutherians. In addition, the microarray data showed decreased expression of *Cd99*, *Shox2 *and *Csf2ra*, that also lie within the pseudoautosomal region in most eutherians. Therefore, while GO analysis identified a subset of downregulated genes involved in brain development at both timepoints, a more in depth analysis of downregulated targets revealed that many are orthologs of PAR1 genes residing on the tip of the X and Y chromosomes in most placental mammals. Overall, our transcriptional screen identified six of these genes, constituting approximately half of all PAR1 orthologs discovered in the mouse genome so far. The more intriguing aspect of this finding is that in the mouse, these genes no longer reside within the PAR1 region but have translocated to autosomes (Figure 1). It also identified two potential novel PAR1 orthologs–*Arse *and *Asmtl*–not previously identified in the mouse genome. At E13.5, these genes represent 6 of the 15 most downregulated transcripts identified by microarray analysis. Strikingly, they constitute 4 of the top 5 most downregulated genes in the microarray performed on P0.5 forebrain tissue (*Arse *and *Shox2 *were not significantly decreased in the microarray at P0.5) (Table 1, Additional file 1B). These results suggest that ATRX normally participates in the transcriptional activation of these genes during both the proliferative (E13.5) and more differentiative (P0.5) stages of forebrain development.

**Table 1:** Downregulated genes in the ATRX-null forebrain at E13.5 and P0.5.

		Chromosome			
				
Gene	Description	Mouse	Human	Fold Change	Genbank
**E13.5 Downregulated Genes**					
**IMAGE:354942**	**Similar to dehydrogenase/reductase (SDR family) X chromosome (*Dhrsxy*)**^1^	**4**	**X/Y PAR**	**-4.81**	W45978
***Csf2ra***	**Colony stimulating factor 2 receptor, alpha, low-affinity (granulocyte-macrophage)**	**19**	**X/Y PAR**	**-3.05**	BM941868
*Vit*	Vitrin	17	2	-2.74	AF454755
***Shox2***	**Short stature homeobox 2**	**3**	**X/Y PAR**	**-2.73**	AV332957
*Tcf7l2*	Transcription factor 7-like 2, T-cell specific, HMG-box	19	10	-2.72	BB175494
*Gbx2*	Gastrulation brain homeobox 2	1	2	-2.55	L39770
**IMAGE:3326212**	**Similar to Arylsulfatase E (*Arse*)**^1^	**-**	**X/Y**	**-2.21**	BE457721
*Syt13*	Synaptotagmin 13	2	11	-2.19	BB244585
***Cd99***	**CD99 antigen**	**4**	**X/Y PAR**	**-2.09**	AK004342
*Nxph1*	Neurexophilin 1	6	7	-1.92	BB274960
*Neurod4*	Neurogenic differentiation 4	10	12	-1.86	NM_007501
*Nxph2*	Neurexophilin 2	2	2	-1.86	BB169128
*Peg10*	Paternally expressed 10	6	7	-1.86	BG076799
**RIKEN:1810009N02**	**Similar to *Asmtl *(acetylserotonin O-methyltransferase-like)**^1^	**-**	**X/Y PAR**	**-1.81**	AK007409
*Wif1*	Wnt inhibitory factor 1	10	12	-1.81	BC004048

					

**P0.5 Downregulated Genes**					
***Csf2ra***	**Colony stimulating factor 2 receptor, alpha, low-affinity (granulocyte-macrophage)**	**19**	**X/Y PAR**	**-7.14**	BM941868
*Nr4a2*	Nuclear receptor subfamily 4, group A, member 2	2	2	-3.33	NM_013613
**IMAGE:354942**	**Similar to *Dhrsxy *(dehydrogenase/reductase (SDR family) X chromosome**^1^	**4**	**X/Y PAR**	**-3.33**	W45978
IMAGE:5656844	Unknown EST	-	-	-2.86	BI320076
*Met*	Met proto-oncogene	6	7	-2.78	BG060788
***Cd99***	**CD99 antigen**	**4**	**X/Y PAR**	**-2.22**	AK004342
*Dsc3*	Desmocollin 3	18	18	-2.22	NM_007882
*Mbp*	Myelin basic protein	18	18	-2.17	AI323506
*Cbln4*	Cerebellin 4 precursor protein	2	20	-2.08	AV343573
*EST*	Unknown EST	-	-	-2.08	BI202412
*Trpc4*	Tansient receptor potential cation channel, subfamily C, member 4	3	13	-2.04	BB271442
**RIKEN:1810009N02**	**Similar to *Asmtl *(acetylserotonin O-methyltransferase-like)**^1^	**-**	**X/Y PAR**	**-2.00**	AK007409

^1^By BLASTn

cRNA was generated from total forebrain RNA from three pairs of littermate-matched ATRX-null and wild type forebrain tissue and hybridized to an Affymetrix Mouse Genome 430 2.0 Array. Data was analyzed using GeneSpring. Probesets were filtered by fold change (1.8 fold at P0.5, 2 fold at E13.5) and confidence, P < 0.05, and duplicate genes were removed. Ancestral PAR genes are highlighted in grey.

### Verification of gene expression changes

**Figure 2:**
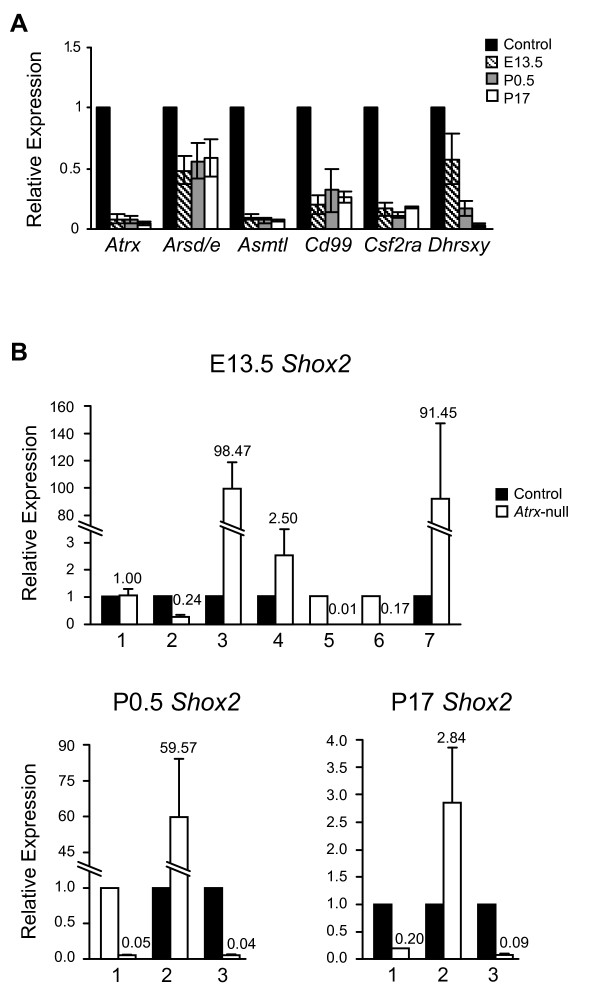
**Relative expression of ancestral PAR genes in ATRX-null mouse forebrains**. (A) Real-time quantitative RT-PCR of the indicated PAR1 genes showed a decrease in expression in the *Atrx*-null mouse forebrain. RNA was isolated from littermate-matched *Atrx*-null and control embryos/mice at E13.5, P0.5 and P17. Results were normalized to β-actin expression levels. Error bars represent standard error of the mean between biological replicates (n = 3). (B) Expression of *Shox2 *in seven (E13.5) or three (P0.5 and P17) littermate-matched pairs. Error bars represent standard error of the mean for technical replicates. In (A) and (B) expression levels for the control forebrains were set to one for each reaction (represented by the black bars).

To validate the microarray results, we performed real-time quantitative reverse transcriptase polymerase chain reaction (qRT-PCR) analysis of *Dhrsxy, Cd99*, *Csf2ra*, *Shox2 *and also the putative new orthologs of *Asmtl *and *Arse *in *Atrx*-null and control E13.5 and P0.5 forebrain (n = 3 at each time point). Since *Arse *and *Asmtl *have not yet been identified in the mouse, we sequenced the PCR products to ensure they corresponded to the transcripts identified on the microarray, and not to other contaminating sequences. The qRT-PCR results confirmed that five of the six genes exhibit decreased expression in the *Atrx*-null forebrain at E13.5, and that these genes remain downregulated at P0.5 (Figure 2A). In addition, analysis at P17 demonstrated decreased expression of ancestral PAR genes at this later time point as well (Figure 2A). One exception was *Shox2 *which exhibited highly variable expression differences between the *Atrx*-null and control tissue at E13.5, P0.5 and P17, ranging from a 170 fold decrease to a 90 fold increase (Figure 2B). Therefore, while the expression of *Shox2 *is clearly affected by the loss of ATRX protein, the outcome on expression levels appears to be highly variable and does not validate the consistent downregulation observed by microarray analysis.

Our discovery that the expression of several ancestral PAR1 genes is controlled by ATRX throughout the early developmental period of the mouse brain reveals an unexpected association between the levels of ATRX protein and the expression of these ancestral PAR1 genes.

### Identification of a novel *ARS *family mouse homolog

In humans, a cluster of *ARS *genes are located approximately 115 kilobases centromeric to the PAR1 region on the X chromosome, but still possess the ability to escape XCI in females [23]. Located outside the PAR1, these genes do not have an identical homolog on the Y chromosome but have pseudogenes, and in the evolutionary past it is believed that they were true pseudoautosomal genes with identical copies on both the X and Y chromosome [24].

A multiple alignment of amino acid sequences of [GenBank:BE45772] suggested that it is a fragment of the full length ARSE protein, aligning in the middle of the approximately 600 amino acid ARSE protein of multiple other species (Additional file 3). The putative mouse ARSE is 65% identical to rat and 47% identical to human.

**Figure 3:**
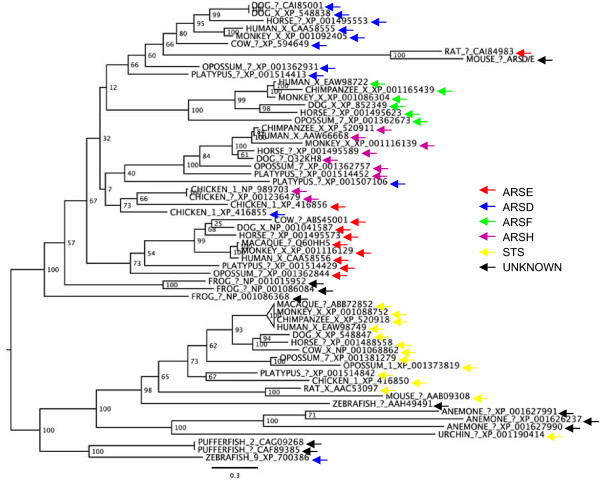
**Phylogenetic tree of ARS proteins**. Human ARSE [GenGank:NP_000038] was used as a seed to search the GenBank NR database for orthologs and an approximate maximum-likelihood tree was generated. The putative ARS family gene downregulated in the *Atrx*-null forebrain clusters closely with rat *ARSE *but with the ARSD proteins. Entries are annotated with species, chromosome (where known) and GenBank Accession number.

Phylogenetic analysis demonstrated that the mouse ARSE sequence clusters with near certainty with the rat ARSE, however, this putative ARSE clustered within the ARSD proteins, not ARSE as expected (Figure 3). Therefore, we propose that we have identified a member of the PAR1 ARS family but at this time cannot determine the exact identity and will refer to this sequence as *Arsd/e*. We note that the long branch-length between the rodent ARS sequences and the remaining ARSD clade may be an artifact due to the short mouse sequence and its high similarity to the rat sequence, which has undergone seemingly accelerated evolutionary change.

Comparisons to available mouse *Ars *gene family members shows that [GenBank:BE457721] is more similar to *Arse *genes in rat than to other mouse arylsulfatase family members (Additional file 4), suggesting that we have identified *Arse*. This data, combined with our ability to specifically amplify this transcript from mouse brain cDNA and also from a commercially available E15 cDNA library (data not shown), indicates that we have likely identified the mouse homologue of a previously unidentified mouse *Ars *gene rather then a gene fragment from a known mouse family member.

**Figure 4:**
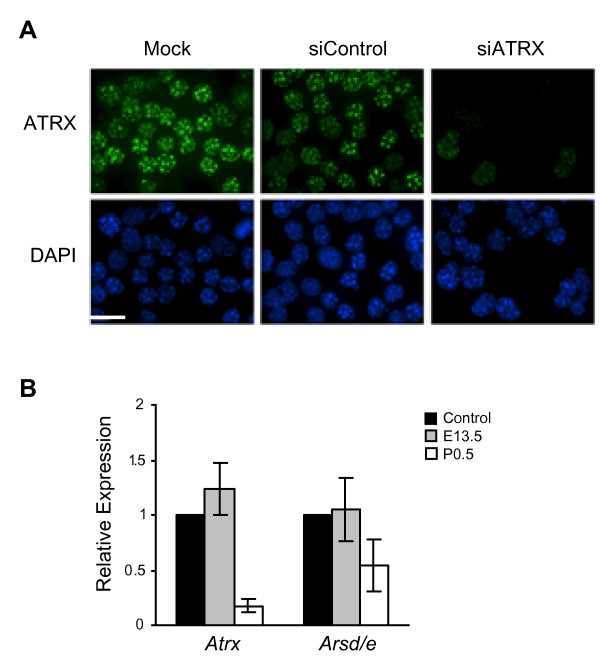
***Arsd/e *transcriptional downregulation is recapitulated in ATRX-depleted cells**. (A) RNA interference was used to deplete ATRX in Neuro-2a neuroblastoma cells. Cells were transfected with 8 nM siRNA, fixed after 72 h and processed for immunofluorescence staining using an anti-ATRX primary antibody (H300) and anti-rabbit Alexa 488 secondary antibody, then counterstained with DAPI to detect nuclei. In the siATRX treated samples, approximately 95% of cells were negative for ATRX. Scale bar = 20 μM. (B) Total RNA was isolated for quantitative real-time PCR of *Atrx *and *Arsd/e *gene expression at 72 hours post-transfection. Mock (transfection reagent only) expression levels were set to one and a non-specific siRNA was used as a control. Results were normalized to β-actin expression levels. Error bars represent standard error of the mean (n = 3).

To further confirm the identity of [GenBank:BE457721], we assessed the outcome of ATRX depletion on *Arsd/e *expression by RNA interference in the Neuro-2a cultured neuroblastoma cell line. Small interfering RNAs (siRNAs) were used to transiently deplete ATRX, as was done previously [25]. Cells transfected with a non-specific siRNA or no siRNA ("Mock") were used as controls. At 72 hours following siRNA transfection, we monitored the effectiveness of ATRX depletion by indirect immunofluorescence using an ATRX-specific antibody (H300) and qRT-PCR analysis of *Atrx *expression levels using primers that simultaneously amplify both the full length isoform and the reported truncated isoform [26]. In the siATRX-treated samples, approximately 95% of cells were negative for ATRX (Figure 4A) and *Atrx *transcript levels were depleted by approximately 5 fold (Figure 4B). We then used qRT-PCR to determine the outcome of ATRX silencing on the expression level of the *Arsd/e*. Similar to the results obtained in the *Atrx*-null forebrain, the expression of *Arsd/e *was decreased two fold (Figure 4B). These findings support that we have identified the mouse *Arsd/e *and confirm the regulation of this ancestral PAR gene by ATRX, and that this outcome on gene expression can be recapitulated in two different systems: *in vivo *in the ATRX-null developing forebrain and *in vitro *in ATRX-depleted cultured neuronal cells.

### Identification of an *ASMTL*-like gene

[GenBank:AK007409] is the RIKEN cDNA 1810009N02 gene and contains a musculoaponeurotic fibrosarcoma (MAF) domain. A multiple sequence alignment of amino acid sequences was used to further determine the identity of [GenBank:AK007409] (Additional file 5). [GenBank:AK007409] aligns to the N terminus of ASMTL from multiple other species. The N terminal portion of ASMTL also contains a MAF domain. Human *ASMTL *was generated by a fusion of a duplicated acetylserotonin O-methyltransferase (*ASMT*) with the bacterial *maf *gene [27]. While [GenBank:AK007409] contains a MAF domain, it lacks the ASMT domain. However, this is similar to the putative rat ASMTL (Accession [GenBank:NP_001099385]) which also lacks the ASMT domain. The putative mouse ASMTL is 54% identical to rat, and 51% identical to the human protein.

**Figure 5:**
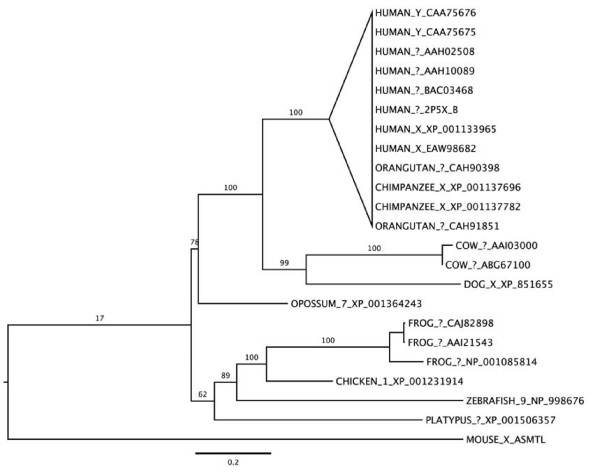
**Phylogenetic tree of ASMTL proteins**. Human ASMTL [GenBank:NP_004183] was used as a seed to search the GenBank NR database for orthologs and an approximate maximum-likelihood tree was generated. The putative mouse ASMTL lacks the ASMT domain and clusters well outside the mammalian clade, indicating that it has considerably diverged compared to the putative ancestor. Entries are annotated with species, chromosome (where known) and GenBank Accession number.

In contrast to ARSE, ASMTL has fewer discernable high-similarity full-length orthologs, and its evolution appears tied to the pseudoautosomal region [27]. Therefore fewer sequences were available for analysis. Figure 5 shows the inferred phylogeny of the ASMTL family, with the primate branches collapsed for clarity. With fairly high bootstrap support, the tree mirrors the known branching of the placental mammals, marsupials, monotremes, birds, amphibians, and fish. The mouse sequence displays the only anomalous placement in the tree, clustering well outside the mammalian clade. Both the placement and the branch-length of the mouse sequence indicate that it is of considerably evolutionarily derived character compared to the putative ancestor, and it appears to have followed an evolutionary path quite different from its paralogs. The lack of the ASMT domain in the mouse sequence may also be responsible for the placement of the mouse sequence in the tree. As with ARSE, some of this divergence may be due to the availability of a partial mouse sequence, but the sequence remains quite unique, nonetheless.

## Discussion

Mutations in the *ATRX *gene result in profound cognitive deficits, facial dysmorphisms, as well as skeletal and urogenital abnormalities [15]. Global deletion of *Atrx *in mouse embryonic stem cells results in a growth disadvantage [28], and conditional loss of *Atrx *beginning at the 8–16 cell stage leads to embryonic lethality by E9.5 [28]. To bypass early embryonic lethality, we have previously used a conditional approach to delete *Atrx *in the mouse forebrain beginning at E8.0. These mice have significantly increased cortical progenitor cell apoptosis, causing a reduction in forebrain size and hypocellularity in the neocortex and hippocampus [19]. ATRX is a chromatin remodeling protein [16] and has been proposed to regulate gene expression by modulating chromatin structure, but gene targets of ATRX have not yet been reported. We used a microarray approach to perform large-scale analysis of gene expression changes in the ATRX-null versus wild type mouse forebrain at E13.5 and P0.5. The fact that relatively few genes display altered expression indicates that ATRX is not a global regulator of gene expression but likely controls specific gene loci. It is not clear at this point if ATRX acts by binding directly to DNA or through other unidentified factors to upregulate the ancestral PAR genes identified in our study. The only target of ATRX identified to date is α globin which is downregulated in patients with germline or somatic *ATRX *mutations [29], including α-thalassemia myelodysplastic syndrome [30], although evidence that ATRX directly binds to the α globin locus is still lacking.

Through global transcriptional profiling we have now identified a distinct group of genes, the ancestral PAR genes, that are controlled by ATRX in the mouse brain. The human PAR1 contains 24 genes, but only 10 of these have been reported in the mouse genome. *Arsd/e*, *Asmtl*, *Cd99*, *Csf2ra*, *Dhrsxy *and *Shox2 *were among the most downregulated genes identified in the ATRX-null embryonic forebrain. Although these genes are unrelated in function, they share a common ancestral location in the PAR1 of the X chromosome millions of years ago. Our findings demonstrate that they have maintained a mechanism of co-regulation that was conserved in evolution and that requires ATRX, even after their dispersal to autosomes in the mouse genome.

The PAR1 region exhibits recombination rates approximately 10 times higher than the rest of the human genome [8]. Consequently, genes in this region undergo rapid evolution leading to high interspecies divergence [9,31] making positive identification of homologs difficult. Using multiple sequence alignments and phylogenetic analysis we have identified *Arsd/e *and *Asmtl *as putative novel mouse ancestral PAR transcripts. Identity between mouse and human sequences are 47%, 40% and 51% for *ARSE *[GenBank:NM_000047], *ARSD *[GenBank:NM_001669] and *ASMTL *[GenBank:NM_004192], respectively, which is similar to what was reported for other PAR1 genes. For example, *DHRSXY *exhibits 59% protein identity between humans and mice [9], *CD99 *46% identity [32], and 35% for *CSF2RA *[33].

*ARSD *and *ARSE *are members of the arylsulfatase gene family and are located just outside the human PAR1 in a cluster of four arylsulfatase genes [24]. *ARSE *gene mutations cause X-linked chondrodysplasia punctata, a disorder characterized by abnormalities in cartilage and bone development [34]. ARSE may therefore play a role in the skeletal defects seen in patients with the ATR-X syndrome if it is also regulated by ATRX in humans. The role of *ARSD *is unknown and it has no demonstrated sulfatase activity despite its high conservation of the N-terminal domain important for catalytic sulfatase activity [35]. *ARSE *exhibits a restricted pattern of expression [23] while *ARSD *is ubiquitously expressed [36].

The function of human *ASMTL *is unknown. The gene was generated by the duplication of the PAR1 gene *Asmt *which then fused with the bacterial *orfE/maf *gene [27]. While other *ASMT *genes involved in the serotonin/N-acetylserotonin/melatonin pathway are expressed specifically in the human brain, pineal gland and retina [37], *ASMTL *has a wider expression pattern and may not be involved in this pathway but could still have methyltransferase activity since it retains the necessary domain [27].

We have also identified the mouse *Shox2 *gene as a potential target of ATRX, and we observed that *Shox2 *expression levels are highly sensitive to ATRX deficiency in the developing mouse brain. Two *SHOX *genes, *SHOX *and *SHOX2 *have been identified in the human genome, on chromosomes X and 3, respectively. Only one mouse homolog has been identified and is mapped to chromosome 3. Like *ARSE*, *SHOX *genes are involved in skeletal development: mutations and deletions in *SHOX *lead to Leri-Weill dyschondrosteosis [38,39] and non-syndromic idiopathic short stature [40,41], and deletions cause the short stature phenotype seen in Turner syndrome [41,42]. SHOX2 is involved in craniofacial and limb development [43] and *SHOX2 *mutations lead to cleft palate [44]. Along with *ARSE*, the *SHOX *genes provide an intriguing correlation with the skeletal phenotype of ATR-X patients, and future work should address whether these genes are regulated by ATRX in humans.

## Conclusion

**Figure 6:**
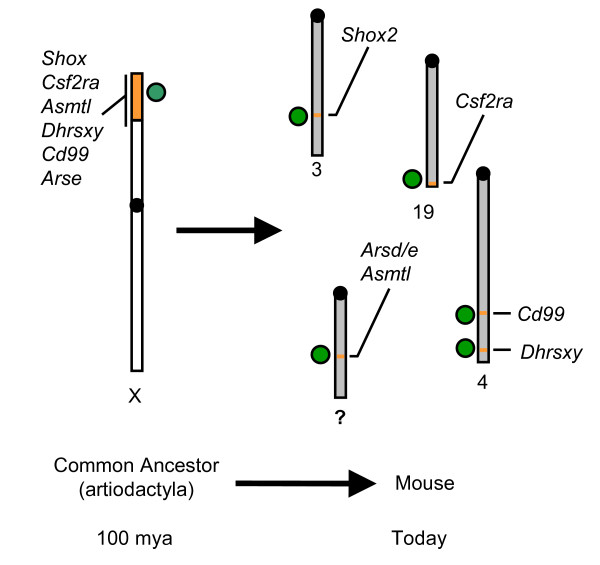
**Proposed model for the co-regulation of ancestral PAR genes by ATRX**. Mice and humans diverged from a common ancestor approximately 50 million years ago. In humans, genes have remained in the PAR region (orange) while in mice and rats they have translocated to autosomes (grey). Our data suggest that despite the translocation of these genes to autosomes (numbered), they still share a common sequence or chromatin environment that allows ATRX (green circles) to keep these genes active. We propose that this common feature was conserved upon translocation from the pseudoautosomal region on the X and Y chromosomes in the ancestral PAR to their current autosomal locations in the mouse genome. mya, millions of years ago.

Collectively, our findings suggest that even though they are now located on different chromosomes, a large subset of ancestral PAR genes might share a common sequence or factor that was conserved upon translocation from the pseudoautosomal region on the X chromosome to their current autosomal locations in the mouse genome (Figure 6). Uniform regulation of gene expression may be due to similar regulatory features such as common sequences or epigenetic modifications (e.g. CpG islands). Despite the sequencing of the human X chromosome, gaps remain, most notably in the PAR1 region [45]. The repetitive nature of the PARs likely explains the paucity of sequence data for these regions, and the lack of genomic sequence data for the PAR1 genes that have translocated to autosomes in the mouse. However, we speculate that ATRX could be targeted to repetitive sequences surrounding these genes. One indication that ATRX would preferentially target repetitive sequences comes from studies done in human ATR-X syndrome patients. The analysis of blood samples revealed altered DNA methylation of several highly repeated sequences including ribosomal DNA arrays, the Y-specific repeat DYZ2 and subtelomeric repeats [18]. Conservation of repetitive elements in the PAR1 region of eutherians may have been maintained with the PAR1 genes as they moved to autosomes, and perhaps allow ATRX to target these genes in their modern chromosomal locations.

Future work should focus on identifying the molecular mechanisms by which ATRX can co-regulate this diverse set of genes linked by their ancestral localization in the PAR1 region. This will lead to a better understanding of ATRX function in the regulation of chromatin structure and its effects on gene expression in general.

## Methods

### Mouse husbandry

Mice conditionally deficient for ATRX in the forebrain were generated by crossing *Atrx*^*loxP *^females with heterozygous *Foxg1Cre *male mice, as previously described [19]. Pregnant females were sacrificed at E13.5, embryos were recovered and yolk sac DNA was genotyped by PCR using the primers 17F, 18R and *neo*^*r*^as described previously [19]. For newborns (P0.5) and juveniles (P17), pups were sacrificed and tail DNA was used for genotyping as previously described [19].

### Microarray analysis

Total forebrain RNA (10 μg) was isolated from three E13.5 and four P0.5 pairs of littermate-matched ATRX-null and control embryos using the RNeasy Mini kit (Qiagen). cRNA was generated and hybridized to an Affymetrix Mouse Genome 430 2.0 Array at the London Regional genomics Center (London, Canada). For the analysis at E13.5, RNA from two forebrains was pooled for each array. Probe signal intensities were generated using GCOS1.4 (Affymetrix Inc., Santa Clara, CA) using default values for the Statistical Expression algorithm parameters and a Target Signal of 150 for all probe sets and a Normalization Value of 1. Gene level data was generated using the RMA preprocessor in GeneSpring GX 7.3.1 (Agilent Technologies Inc., Palo Alto, CA). Data were then transformed (measurements less than 0.01 set to 0.01), normalized per chip to the 50^*th *^percentile, and per gene to control samples. Probe sets representing *Atrx *transcripts were removed (10 sets). Remaining probe sets were filtered by fold change of either ≥1.5 or 2 between control and *Atrx*-null samples, and by confidence level of P < 0.05. Heatmaps were generated using the GeneSpring hierarchical clustering gene tree function. Significantly overrepresented GO categories were determined using GeneSpring: at E13.5 and P0.5, probe sets were filtered by 1.5 fold change, P < 0.05 and categorized as either up or downregulated. Where there were multiple probe sets for a gene, duplicates were removed. P < 0.001 was used as the significance cutoff.

### qRT-PCR

Total RNA was isolated using the RNeasy Mini kit (QIAGEN). First-strand cDNA was synthesized from 3 μg of total RNA using the SuperScript™ II Reverse Transcriptase kit (Invitrogen) with 25 mM dNTPs (GE Healthcare), 1 μL porcine RNAguard (GE Healthcare) and 3 μL random primers (GE Healthcare). PCR reactions were performed on a Chromo4 Continuous Fluorescence Detector in the presence of iQ™ SYBR Green Supermix and recorded using the Opticon Monitor 3 software (Bio-Rad Laboratories, Inc.). Samples were amplified as follows: 95°C for 10 seconds, annealed for 20 seconds, 72°C for 30 seconds (See Additional file 6 for primer sequences and annealing temperatures). After amplification a melting curve was generated, and samples were run on a 1.5% agarose gel (75 V for 1 h) to visualize amplicon purity. Standard curves were generated for each primer pair using three fold serial dilutions of control cDNA. Primer efficiency was calculated as E = [10^(-1/slope)-1^]*100, where a desirable slope is -3.32 and r^2 ^> 0.99. Samples were normalized to β-actin expression and relative gene expression levels were calculated using GeneX software (Bio-Rad Laboratories, Inc.).

For *Arsd/e *and *Asmtl*, the PCR products were gel extracted using the QIAquick Gel Extraction Kit (QIAGEN) according to the manufacturer's instructions and sequenced at the DNA Sequencing Facility at Robarts Research Institute (London, Canada).

### Bioinformatics analysis of novel ancestral PAR genes

Probeset sequences were obtained from the Netaffx website  and used for BLASTn searches . For calculation of interspecies similarity, sequences were obtained from NCBI RefSeq  or Ensemble  where RefSeq sequences were not available, and pairwise comparisons made using Jalview [46].

For generation of trees and sequence alignments, human ARSE (SwissProt P51690, RefSeq NP_000038) and human ASMTL (SwissProt O95671, RefSeq NP_004183) were used as seeds and the GenBank NR database was searched for high-similarity, full-length orthologs and paralogs. Fifty-nine ARSE and twenty-two ASMTL sequences met or exceeded the similarity cutoff, with resultant species spanning the metazoa from anemone and urchin to a diverse set of vertebrates. Sequences were aligned using T-Coffee 5.56 [47] using default parameters. Alignments were manually adjusted via inspection prior to further analysis. Approximate maximum-likelihood trees were built using PHYML 2.4.5 [48] using the WAG model of protein evolution [49] and a seven-category Gamma-plus-invariant model of rate heterogeneity. All rate parameters were estimated from the data. One hundred bootstrap replicates were performed to assess support for the inferred tree topology. All trees are presented as midpoint-rooted phylograms. Since the given mouse sequences were quite short compared to the full protein length, two sets of trees were built for each family to assess if the mouse sequences were long enough to definitively support their taxonomic clustering. One set utilized a "trimmed" alignment where all alignment columns outside the mouse sequence domain were removed. The trees produced with this trimmed alignment were compared with the set of trees produced from the alignment of the mouse sequences to their respective full-length proteins. For both ARSD/E and ASMTL, very little difference was observed between full-length and trimmed-alignment trees. The trimmed alignments tended to exaggerate sequence divergence and modestly lower bootstrap support levels. Overall topology did not appear significantly different, however, and the text references the full-length sequence phylogeny exclusively.

### Cell culture and RNA interference

Neuro-2a cells were grown at 37°C with 5% CO_2 _in EMEM supplemented with 10% fetal bovine serum (Sigma-Aldrich). For siRNA treatment, 1.5 × 10^4 ^cells were plated in a plastic six well dish (Corning Incorporated) on glass coverslips and allowed to grow to 15% confluency (approximately 24 hours). Cultures were transfected using Lipofectamine 2000 (Invitrogen) with 8 nM siATRX (Dharmacon), a non-specific control siRNA (Sigma-Aldrich), or with no siRNA ("Mock") according to the manufacturers' instructions (for siRNA sequences refer to [25]). Total RNA was extracted from cells after 72 hours, cDNA was generated and qPCR analysis performed as described above. Alternatively, cells were processed for immunofluorescence staining as described below.

### Immunofluorescence

Neuro-2a cells were fixed using 3:1 methanol:ethanol, incubated for 1 h with the primary antibody (H300 anti-ATRX, 1:100 dilution; Santa Cruz) followed by the secondary antibody (goat-anti rabbit Alexa 594, 1:1500 dilution; Molecular Probes), then counterstained with 4',6-diamidino-2-phenylindole (DAPI) (Sigma-Aldrich) for 5 min. Coverslips were mounted with Vectashield (Vector Laboratories), Z-stack images were captured using a Leica DMI6000b inverted microscope and Openlab software (v5.0, Improvision) and processed using Volocity software (v4.0, Improvision); deconvolution was performed using iterative restoration set with a confidence limit of 95%.

## Abbreviations

PAR: Pseudoautosomal region; SWI/SNF: Switching/Sucrose non-fermenting; ATRX: α thalassemia mental retardation: X linked; XCI: X chromosome inactivation; Csf2ra: Colony stimulating factor 2 receptor: alpha; Cd99: CD99 antigen; Dhrsxy: dehydrogenase/reductase (SDR family) X chromosome; Ars: Arylsulfatase; Asmtl: acetylserotonin O-methyltransferase-like; ADD: Atrx Dnmt3a/b Dnmt3L; rDNA: ribosomal DNA; Foxg1: forkhead box G1; Cre: cyclization recombinase; EST: Expressed Sequence Tag; BLASTn: Basic Local Alignment Search Tool nucleotide; BLAT: BLAST-like Alignment Tool; E: embryonic; P: postnatal; Sts: steroid sulfatase; siRNA: Small interfering RNA; RT-PCR: reverse-transcriptase polymerase chain reaction; MAF: musculoaponeurotic fibrosarcoma; ASMT: acetylserotonin O-methyltransferase; SDR: Short-chain dehydrogenase/reductase; SHOX: short stature homeobox; ATR-X: alpha thalassemia mental retardation: X linked (referring to the syndrome); Neuro-2a: Neuroblastoma-2a; DAPI: 4',6-diamidino-2-phenylindole; GO: Gene Ontology.

## Authors' contributions

ML performed animal husbandry, isolated RNA from E13.5 forebrains for microarray and qRT-PCR analysis, analyzed microarray results, carried out qRT-PCR analysis, cell culture and immunofluorescence, initial bioinformatics analysis and wrote the manuscript. AF performed the phylogenetic analysis of *Arsd/e *and *Asmtl *and provided expertise on the bioinformatics analysis. DT performed animal husbandry and extracted RNA from P0.5 forebrains for microarray and qRT-PCR analysis. CS performed animal husbandry and provided technical expertise. NB provided technical expertise, intellectual direction and assisted with the writing of the manuscript.

## Supplementary Material

Additional file 1 **Summary of microarray results**. cRNA was generated from total forebrain RNA from three pairs of littermate-matched ATRX-null and wild type forebrain tissue and hybridized to an Affymetrix Mouse Genome 430 2.0 Array. Data was analyzed using GeneSpring. Probe sets were filtered by fold change (1.5 and 2 fold at E13.5 and P0.5) and confidence, P < 0.05, and duplicate genes were removed. (A) Venn diagrams to categorize probe sets according to developmental timepoint and fold change in expression levels. (B) Hierarchical clustering of differentially expressed probe sets. Approximately two-thirds of the misregulated genes are upregulated. Ancestral PAR genes are consistently downregulated at both timepoints and are indicated by blue text. Probe sets were filtered by 1.5 fold or 2 fold change, P < 0.05, at either E13.5 or P0.5. Normalized expression levels are displayed. Click here for file

Additional file 2 **Significantly misregulated GO categories**. GeneSpring was used to identify significantly overrepresented GO categories. Probe sets were filtered by 1.5 fold change, P < 0.05 and categorized as either up or downregulated. When there were multiple probe sets for a gene, duplicates were removed. P < 0.001 was used as the significance cutoff. Click here for file

Additional file 3 **Amino acid alignment of a small portion of ARSD/E between multiple species**. Sequences were aligned using T-Coffee 5.56 [47] using default parameters, edited using JalView [46] and shaded using Boxshade [51]. Mouse ARSD/E has highest identity to rat ARSE (65%). Accession numbers are ARSE: chicken [GenBank:XP_416856], cow [GenBank:ABS45001], dog [GenBank:NP_001041587], horse [GenBank:XP_001495573], macaque [GenBank:Q60HH5], human [GenBank:CAA58556], platypus [GenBank:XP_001514429], opossum [GenBank:XP_001362844], pufferfish [GenBank:CAG09268], rat [GenBank:CAI84983]. ARSD: dog [GenBank:XP_548838], horse [GenBank:XP_001495553], human [GenBank:CAA58555], macaque [GenBank:XP_001092405], opossum, [GenBank:XP_001362931], platypus [GenBank:XP_001507106], chicken [GenBank:XP_416855], zebrafish [GenBank:XP_700386]. Mouse *Arsd/e *translated from [GenBank:BE457721]. Click here for file

Additional file 4 **Comparisons of Ars family members**. The transcript identified as a putative mouse *Arse *ortholog is more similar to rat *Arse *then to any other *Ars *family members. Pairwise percent identities were calculated using Jalview [46]. Accession numbers are: *Arse *[GenBank:BE45772], *Arsa *[GenBank:NM_009713], *Arsb *[GenBank:NM_009712.3], *Arsc/Sts *[GenBank:NM_009293.1], *Arsg *[GenBank:NM_028710.2], *Arsi *[GenBank:NM_001038499.1], *Arsj *[GenBank:NM_173451.2], Arsk [GenBank:NM_029847.4], rat *Arse *[GenBank:NM_001047885.1], rat *Arsc/Sts *[GenBank:NM_012661.1]. Click here for file

Additional file 5 **Amino acid alignment of the N terminal of ASMTL between multiple species**. Sequences were aligned using T-Coffee 5.56 [47] using default parameters, edited using JalView [46] and shaded using Boxshade [51]. The putative mouse ASMTL aligns within the N terminal MAF domain and is most similar to rat ASMTL (54% identity) which also contains only the MAF domain. Accession numbers are: human [GenBank:XP_001133965], orangutan [GenBank:CAH90398], chimpanzee [XP_001137696], cow [GenBank:AAI03000], dog [GenBank:XP_851655], frog [GenBank:NP_001085814], chicken [GenBank:XP_001231914], zebrafish [GenBank:NP_998676], platypus [GenBank:XP_001506357], mouse [GenBank:NP_081215]. Click here for file

Additional file 6 **Conditions for quantitative real-time PCR**. Primer sequences and annealing temperatures used for quantitative real-time PCR confirmation of downregulated ancestral PAR genes. Click here for file
