# Supplementary figures and images for: The SWI/SNF protein ATRX co-regulates pseudoautosomal genes that have translocated to autosomes in the mouse genome
Source: BMC Genomics. 2008 Oct 8;9:468. doi: 10.1186/1471-2164-9-468 (PMC2577121; doi:10.1186/1471-2164-9-468)

A

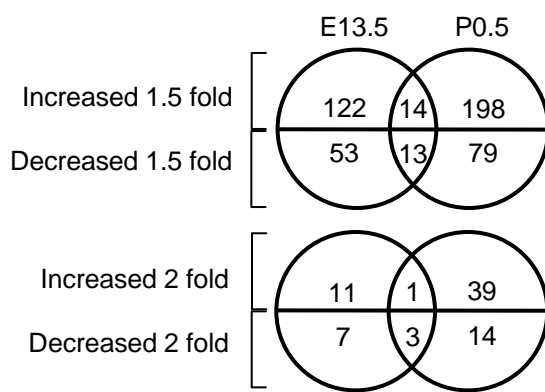

B

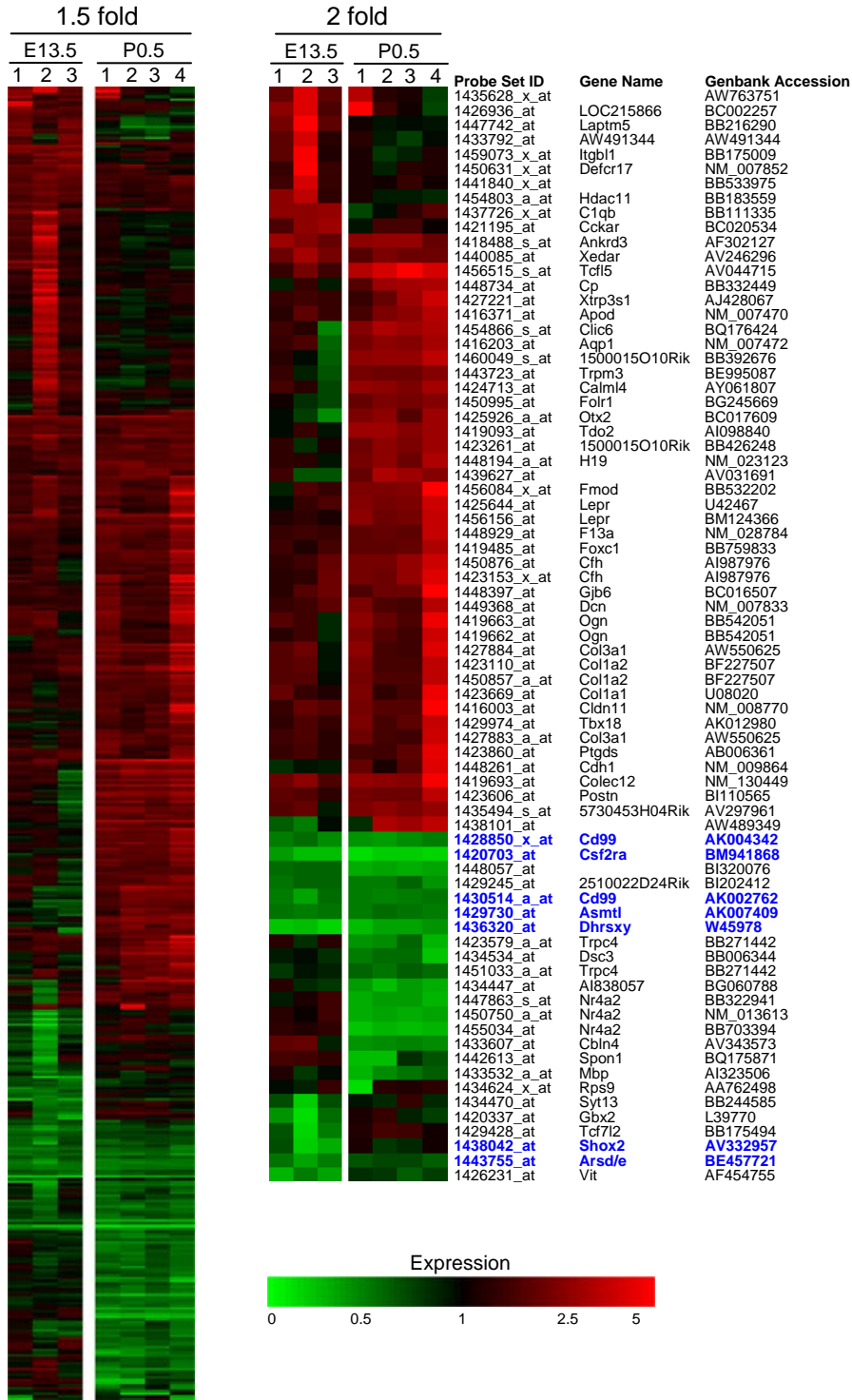

Supplement: Additional file 1 — Summary of microarray results. cRNA was generated from total forebrain RNA from three pairs of littermate-matched ATRX-null and wild type forebrain tissue and hybridized to an Affymetrix Mouse Genome 430 2.0 Array. Data was analyzed using GeneSpring. Probe sets were filtered by fold change (1.5 and 2 fold at E13.5 and P0.5) and confidence, P < 0.05, and duplicate genes were removed. (A) Venn diagrams to categorize probe sets according to developmental timepoint and fold change in expression levels. (B) Hierarchical clustering of differentially expressed probe sets. Approximately two-thirds of the misregulated genes are upregulated. Ancestral PAR genes are consistently downregulated at both timepoints and are indicated by blue text. Probe sets were filtered by 1.5 fold or 2 fold change, P < 0.05, at either E13.5 or P0.5. Normalized expression levels are displayed. [file 1471-2164-9-468-S1.pdf]
