# Supplementary material for: The SWI/SNF protein ATRX co-regulates pseudoautosomal genes that have translocated to autosomes in the mouse genome
Source: BMC Genomics. 2008 Oct 8;9:468. doi: 10.1186/1471-2164-9-468 (PMC2577121; doi:10.1186/1471-2164-9-468)
Supplement: Additional file 3 — Amino acid alignment of a small portion of ARSD/E between multiple species. Sequences were aligned using T-Coffee 5.56 [47] using default parameters, edited using JalView [46] and shaded using Boxshade [51]. Mouse ARSD/E has highest identity to rat ARSE (65%). Accession numbers are ARSE: chicken [GenBank:XP_416856], cow [GenBank:ABS45001], dog [GenBank:NP_001041587], horse [GenBank:XP_001495573], macaque [GenBank:Q60HH5], human [GenBank:CAA58556], platypus [GenBank:XP_001514429], opossum [GenBank:XP_001362844], pufferfish [GenBank:CAG09268], rat [GenBank:CAI84983]. ARSD: dog [GenBank:XP_548838], horse [GenBank:XP_001495553], human [GenBank:CAA58555], macaque [GenBank:XP_001092405], opossum, [GenBank:XP_001362931], platypus [GenBank:XP_001507106], chicken [GenBank:XP_416855], zebrafish [GenBank:XP_700386]. Mouse Arsd/e translated from [GenBank:BE457721]. [file 1471-2164-9-468-S3.pdf]

```
ARSE
Chicken     428 LQGTVQHSEHEFMFH-YCGVVLHAVRWHQKDR-GTIWKAHYATPLFKPEDSGACFE----
Cow         438 LQGTARHSDHEFLMH-YCESFLHAARWHQRD----LWKVHFTTPIFQPDGAGACYG----
Dog         437 LLGTAQHSDHEFLLH-YCENFLHAARWRQRDG-GRLWKVHYMTPLFHPEGAGACYG----
Horse       435 LRGTARHSDHEFLLH-YCGKFLHAARWHQRDR-GAVWKVHYVTPVFHPDGAGACYG----
Macaque     441 LLGTAQHSDHEFLMH-YCEGFLHAARWHQRDR--TTWKVHFVTPVFQPEGAGACYG----
Human       441 LLGTAQHSDHEFLMH-YCERFLHAARWHQRDR-GTMWKVHFVTPVFQPEGAGACYG----
Platypus    461 LQGTTQRSDHEFLFH-YCETYLHAVRWHHRES-GAVWKVHYVTPVFHPKGAGACYG----
Opossum     435 LWGEIDQSDHEFMFH-YCESYLHAVRWHQKDS-GTVWKVHYVTPIFHPEGSGACYG----
Puffer Fish 504 LEGKVERSEHEFMFH-YCGIYLHAVRWHPPGS-DSVFKVHFFTPNFSPPGAGGCYN----
Rat         443 LRGETWHSAHEVLLH-YCEVFLHAVRWVQRDS-GQVWKAHFVTPTFDPLGSGSCSGAGG-

Mouse         1 LRGEAEHAAHEVLLH-YCEVFLHAARLVQRER-GKVWKVHFVTPTFDPPGSGSCAGPDGG

ARSD
Dog         489 LRGAAEHSAHEFLFH-YCGKYLHAARWHEKDS-GRLWKVHYMTPRFHPKGAGACYG----
Horse       679 LQGAAEHSEHEFLFH-YCGKHLHAARWHDKDS-GRLWKVHYMTPRFHPEGAGACYG----
Human       442 LQGAEARSAHEFLFH-YCGQHLHAARWHQKDS-GSVWKVHYTTPQFHPEERGLLTA----
Macaque     273 LQGAEARSAHEFLFH-YCGQHLHAARWHQRDS-GSVWKVHYTTPQFHPEGAGACYG----
Opossum     399 LQGTVEHSKHHFLFH-YCGIHLHAARWHQKDS-NKIWKVHYITPNFHPEGSGACYG----
Platypus    515 ------HNEFRYLLSAYCLRSTELSTWERRERGEAVWKAHYVTPIFQPAGAGACYG----
Chicken     390 LQGRTQKSEHKFLFH-YCGSYLHAVRWHQKDS-GAVWKAHYVTPNFHPLGAGACYG----
Zebrafish   333 LEGSSSRSQHEFMFH-YCGMYLNAVRWHPQNS-SSIYKVVFFSPVFSPAGSSGCFS----

ARSE
Chicken     482 ------------------RGICPCFGDGVTHHDPPLLFDLSQDPSE
Cow         489 ------------------QIVCPCSGNRVTHHAPPLLFDLSRDPSE
Dog         491 ------------------RGVCPCSGDQVAHHDPPLLFDLSRDPSE
Horse       489 ------------------KAVCPCSGHGVSRHDPPLLFDLSRDPSE
Macaque     494 ------------------RKVCPCFGEKVLHHDPPLLFDLSRDPSE
Human       494 ------------------RKVCPCFGEKVVHHDPPLLFDLSRDPSE
Platypus    515 ------------------RLVCPCFGDRVTHHDPPLLFDLSSDPSE
Opossum     489 ------------------RGVCPCFGDGVIHHDPPLLFDLSKDPSE
Puffer Fish 558 ------------------TRICQCFGKHVSHHDPPLVFDLFRDPSE
Rat         500 -----------------AAAVCPCVGK-VEEHDPPLLFELTSDPGE

Mouse        59 SGGGNDAEAGQEAGAAPQARLCPCVFG-VTQHDPPLLYELTSDPGE

ARSD
Dog         543 ------------------RGVCPCSGDGVTQHSPPLLFELSRDPSE
Horse       733 ------------------QGVCPCSGEGVTQHNPPLLFELSRDPSE
Human       496 ------------------EASAHAEWGGVTHHRPPLLFDLSRDPSE
Macaque     327 ------------------RGVCPCSGEGVTHHRPPLLFDLSRDPSE
Opossum     453 ------------------RGVCPCSGEHVTYHDQPLLFDLSRDPSE
Platypus    565 ------------------SRMCSCSGAGVRRHEPPLLFDLSRDPSE
Chicken     444 ------------------KGICPCFGEGVTHHDPPLLYDLSRDPSE
Zebrafish   387 ------------------TGSCMCHKPHVTYHSPPLVFLISSDPSE
```
