# Supplementary material for: The SWI/SNF protein ATRX co-regulates pseudoautosomal genes that have translocated to autosomes in the mouse genome
Source: BMC Genomics. 2008 Oct 8;9:468. doi: 10.1186/1471-2164-9-468 (PMC2577121; doi:10.1186/1471-2164-9-468)
Supplement: Additional file 5 — Amino acid alignment of the N terminal of ASMTL between multiple species. Sequences were aligned using T-Coffee 5.56 [47] using default parameters, edited using JalView [46] and shaded using Boxshade [51]. The putative mouse ASMTL aligns within the N terminal MAF domain and is most similar to rat ASMTL (54% identity) which also contains only the MAF domain. Accession numbers are: human [GenBank:XP_001133965], orangutan [GenBank:CAH90398], chimpanzee [XP_001137696], cow [GenBank:AAI03000], dog [GenBank:XP_851655], frog [GenBank:NP_001085814], chicken [GenBank:XP_001231914], zebrafish [GenBank:NP_998676], platypus [GenBank:XP_001506357], mouse [GenBank:NP_081215]. [file 1471-2164-9-468-S5.pdf]

```
Mouse         1 MSLTSLALSLRSRRVVLASASPRRKEILGLTGVALEVVPPRFPETLSKAAWPRPQDYARE
Human         1 MVLCPVIGKLLHKRVVLASASPRRQEILSNAGLRFEVVPSKFKEKLDKASFATPYGYAME
Orangutan     1 MVLCPVIGKLLHKRVVLASASPRRQEILSNAGLRFEVVPSKFKEKLDKASFATPYGYAME
Chimpanzee    1 MVLCPVIGKLLHKRVVLASASPRRQEILSNAGLRFEVVPSKFKEKLDKASFATPYGYAME
Cow           1 MLLCPVIGKLQHKRVVLASSSPRRREILSNAGLRFEVVPSRFKEKLHKASFATPQAYAVE
Opossum       1 MVLCPVIRKLVGKRVVLASASPRRQEILNSAGLRFEVVPSRFKETLDKAAFSTPYEYALE
Frog          1 MLLNPVISKLAGKRVVLASASPRRQDILTNVGLRFEVVPSWFKETLDKSVFAAPYEYALE
Chicken       1 MVLSPVLGKLVSKRVVLASASPRRQEILTNVGLRFEVVPSWFKETLEKSSFAAPYEYAIE
Platypus    189 MALSPVIGKLVRKRVVLASASPRRREILSNAGLRFEVVPSKFKETLDKSLFEAPYEYALE

Mouse        61 TATGKAQEVAARLVQEDPETPTIVIGADTVVAVDGRILEKPKDREDALGDLRSLSGKQHQ
Human        61 TAKQKALEVANRLYQKDLRAPDVVIGADTIVTVGGLILEKPVDKQDAYRMLSRLSGREHS
Orangutan    61 TAKQKALEVANRLYQKDLRAPDVVIGADTIVTVGGLILEKPVDKQDAYRMLSRLSGREHS
Chimpanzee   61 TAKQKALEVANRMHQ----------------TVGGLILEKPVDKQDAYRMLSRLSGREHS
Cow          61 TAKQKALEVADRMYQKDLRAPDVVIGADTIVAVGGLILEKPVDKQDAYRMLSRLSGKEHS
Opossum      61 TAKQKALEVANRMNKKDFRSPDVVIGADTIVSVDGQILEKPADKQHAYSMLSRLNGKEHS
Frog         61 TAKQKALEVARRMHMKHLKTPDIVIGADTIVTLEEAILEKPVDKQDAYNMLSRLSGKEHS
Chicken      61 TAKQKALEVANRMHVKHLRTPDIVIGADTIVTVDEQILEKPVDKQDAYRMLSRLSGKEHS
Platypus    249 TAKQKALEVAHRLHRKDFRSPDVVIGADTIVAVEGLILEKPVDKQDAYNMLSRLNGREHS

Mouse       121 VITGVAIVTWG-GCEGPAQ-EVTAFHEETSVTFSPLSEELIREYTDSGEGWDKAGAYAIQ
Human       121 VFTGVAIVHCS-SKDHQLDTRVSEFYEETKVKFSELSEELLWEYVHSGEPMDKAGGYGIQ
Orangutan   121 VFTGVAIVHCS-SKDHQLDTRVSEFYEETKVKFSELSEELLWEYVHSGEPMDKAGGYGIQ
Chimpanzee  105 VFTGVAIVHCS-SKDHQLHTRVSEFYEETKVKFSELSEELLWEYVHSGEPMDKAGGYGIQ
Cow         121 VFTGVAIVHCY-TKDGQLDTGVSEFYEETTVKFSELSEEMLWEYIDSGEPMDKAGGYGIQ
Opossum     121 VFTGVAIVLCS-TKDKQLETEVFEFYEETKVKFSDLSEELLWEYIHSGEPMDKAGGYGIQ
Frog        121 VFTGVSIVLCNRQKDNQLEMDVFDFYEETKVKFADLSEDLLWEYVHSGEPMDKAGGYGIQ
Chicken     121 VFTGVVIIHCS-SKDNQLETEITDFYEETKVKFSDLSEELLWEYIHSGEPMDKAGGYGIQ
Platypus    309 VFTGVAIVHCS-SKDKELTTEVSQFHEETTVKFSELSEELLWDYIHSGEPMDKAGGYGIQ

Mouse       179 ARGAMLVQDVAGDVLNAVGFPLNRFCRELARV--PPT-----------------------
Human       180 ALGGMLVESVHGDFLNVVGFPLNHFCKQLVKLYYPPRPEDLRRSVKHDPIPAADTFEDLS
Orangutan   180 ALGGMLVESVHGDFLNVVGFPLNHFCKQLVKLYYPPRPEDLRRSVKHDPIPAADTFEDLS
Chimpanzee  164 ALGGMLVESVHGDFLNVVGFPLNHFCKQLVKLYYPPRPEDLRRRVKHDSIPAADTFEDLS
Cow         180 ALGGMLVEYVRGDFLNVVGFPLNRFCKELAHLYHGPRAHGAPRQVRHDSIPAVDTFEDLS
Opossum     180 SLGGMLVEYVHGDFLNVVGFPLNHFCKKLVELYYPPS-KDAIHHVKHDSVPSVETFEVLS
Frog        181 SLGGMLVESVHGDFLNVVGFPLNHFCKKLAEIYYPPSKQTINR-IKHDSIPYVETFENLS
Chicken     180 ALGGMLVEYVHGDFLNVVGFPLNHFCKKLAELYCPPLKHTIQH-IKHDSIPSVDTFESLS
Platypus    369 ALGGMLVESVHGDFLNVVGFPLNRFCKQLAALYPAPRL----------PAPPGGTPETLG

Mouse       214 --RTGSEEV---TSQTTGIDSK--------------DVKX
Human       240 DVEGGGSEP---TQRDAGSR--DEKA------------EA
Orangutan   240 DVEGGGSEP---TQRDAGSR--DEKA------------EA
Chimpanzee  224 DVEGGGPEP---AQRDAGSR--DEKA------------EA
Cow         240 DAEGGGSDP---ARAKEGL----EPC------------GV
Opossum     239 DEESGSSNS---TQQNVTSMLKGESAFSCGDPGSLRKKEV
Frog        240 DVEKDCCVKREAPDVKKGSQ--EKCNY-ALRNK----SSN
Chicken     239 DGESESSSFKEHKGAIKLDS--PSGVI-CNSEN----SSG
Platypus    429 REDGPSLPP---L-----------AS------------SD
```
